# The fractional amplitude of low-frequency fluctuations signals related to amyloid uptake in high-risk populations—A pilot fMRI study

**DOI:** 10.3389/fnagi.2022.956222

**Published:** 2022-07-29

**Authors:** Yi-Wen Bao, Yat-Fung Shea, Patrick Ka-Chun Chiu, Joseph S. K. Kwan, Felix Hon-Wai Chan, Wing-Sun Chow, Koon-Ho Chan, Henry Ka-Fung Mak

**Affiliations:** ^1^Department of Diagnostic Radiology, Li Ka Shing Faculty of Medicine, The University of Hong Kong, Hong Kong, Hong Kong SAR, China; ^2^Department of Medicine, Queen Mary Hospital, Hong Kong, Hong Kong SAR, China

**Keywords:** subjective cognitive decline, type 2 diabetes mellitus, Alzheimer's Disease, functional MRI, neural activity, amyloid

## Abstract

**Background:**

Patients with type 2 diabetes mellitus (T2DM) and subjective cognitive decline (SCD) have a higher risk to develop Alzheimer's Disease (AD). Resting-state-functional magnetic resonance imaging (rs-fMRI) was used to document neurological involvement in the two groups from the aspect of brain dysfunction. Accumulation of amyloid-β (Aβ) starts decades ago before the onset of clinical symptoms and may already have been associated with brain function in high-risk populations. However, this study aims to compare the patterns of fractional amplitude of low-frequency fluctuations (fALFF) maps between cognitively normal high-risk groups (SCD and T2DM) and healthy elderly and evaluate the association between regional amyloid deposition and local fALFF signals in certain cortical regions.

**Materials and methods:**

A total of 18 T2DM, 11 SCD, and 18 healthy elderlies were included in this study. The differences in the fALFF maps were compared between HC and high-risk groups. Regional amyloid deposition and local fALFF signals were obtained and further correlated in two high-risk groups.

**Results:**

Compared to HC, the altered fALFF signals of regions were shown in SCD such as the left posterior cerebellum, left putamen, and cingulate gyrus. The T2DM group illustrated altered neural activity in the superior temporal gyrus, supplementary motor area, and precentral gyrus. The correlation between fALFF signals and amyloid deposition was negative in the left anterior cingulate cortex for both groups. In the T2DM group, a positive correlation was shown in the right occipital lobe and left mesial temporal lobe.

**Conclusion:**

The altered fALFF signals were demonstrated in high-risk groups compared to HC. Very early amyloid deposition in SCD and T2DM groups was observed to affect the neural activity mainly involved in the default mode network (DMN).

## Introduction

Alzheimer's Disease (AD) is the most common neurodegenerative disease and is conceptualized as a continuum from the preclinical phase to the clinical stage of dementia (Sperling et al., 2011). Subjective cognitive decline (SCD) is served as a typical preclinical manifestation of AD. Patients with SCD have a 4.5–6.5 times higher risk to proceed into mild cognitive impairment (MCI) or AD dementia in the future, compared to normal elderlies (Jessen et al., 2014b; Rönnlund et al., 2015; Chen et al., 2020). Type 2 diabetes mellitus (T2DM) as a metabolic disorder typically begins at an older age, associated with cognitive impairment with growing evidence (van den Berg et al., 2009; McCrimmon et al., 2012; Vagelatos and Eslick, 2013). Patients with T2DM are proved to show an increased risk of developing AD with a relative risk of 1.46 (Cheng et al., 2012). Additionally, the decline in multiple cognitive domains of T2DM subjects, such as motor function, executive function, and processing speed, is found (Dufouil et al., 2005; Glodzik-Sobanska et al., 2007; Reisberg et al., 2010). The hypometabolism for glucose and decreased resting cerebral blood flow (CBF) in AD have been observed by assessing neuroimaging techniques such as 18F-Fluorodeoxyglucose positron emission tomography (18F-FDG PET; Besson et al., 2015) and arterial spin labeling magnetic resonance (ASL MR) perfusion (Haller et al., 2016). Compared to healthy controls, T2DM, SCD, and AD groups demonstrate significantly decreased CBF found in our previous work (Chau et al., 2020). Furthermore, a negative correlation between chronic hyperglycemia and CBF in non-demented T2DM indicates a link between diabetes and dementia (Chau et al., 2020). As suggested by Sperling et al. (2011), this long preclinical phase may aid in an earlier therapeutic intervention of AD. Therefore, T2DM and SCD as the high-risk population may be the best stages to study AD.

Non-invasive resting-state functional magnetic resonance imaging (rs-fMRI) is introduced to investigate the functional change in neurodegenerative impairment by detecting spontaneous activity. The measurements include functional connectivity (FC; Greicius et al., 2003; Ebisch et al., 2011), regional homogeneity (ReHo; Zang et al., 2004) and amplitude of low-frequency fluctuations (ALFF) /fractional ALFF (fALFF; Fox and Raichle, 2007). The abnormalities of brain function assessed by rs-fMRI were found in AD and MCI compared to healthy controls based on previous research work (He et al., 2007; Wang et al., 2011; Zhang et al., 2012; Zhao et al., 2014). It is also able to document neurological involvement in SCD (Chen et al., 2020; Zhang et al., 2021) and T2DM (Xia et al., 2017) from the aspect of brain dysfunction. As far as we know, only a few studies applied ALFF/fALFF measurements to assess alterations of brain function in T2DM (Xia et al., 2013; Cui et al., 2014; Wang et al., 2014) and SCD (Yang et al., 2018). The manifestation of regional brain function in T2DM and SCD requires more investigation.

Accumulation of amyloid-β (Aβ) starts decades ago before the onset of clinical symptoms (Jack et al., 2010; Sperling et al., 2014). As suggested by previous studies, compared to cognitively normal elderlies (Kang et al., 2017) and SCD (Li et al., 2021) with negative Aβ retention, those with positive results indicated altered regional functional synchronization. Therefore, non-demented participants with abnormal neocortical Aβ pathology demonstrate the changed brain function that provides insight into exploring the relationship between neuropathology and other events such as neurophysiology at a very early stage. Interestingly, in Hahn et al. (2019) study, for cognitively unimpaired elderlies with negative/normal amyloid-PET results, the earliest Aβ deposition in the regions mainly involved in default mode network (DMN) was observed to be associated with enhanced dynamic functional connectivity as well. The regional neural activity could be affected by amyloid status (positive vs. negative) to some degree. Meanwhile, the very early Aβ accumulation in cognitively normal participants with normal amyloid-PET results may already influence brain connectivity, structure, and function. To date, the association between Aβ deposition and local neural activity measured by ALFF/ fALFF, in the cognitively normal elderly, is much less discussed than it should be since it is crucial to understanding the pathophysiological link between the two events.

In this study, we would like to compare the different patterns of fALFF maps between cognitively normal high-risk groups (SCD and T2DM) and healthy elderlies and evaluate the association between fALFF signals and local amyloid deposition in those clusters showing altered neural activity further. In addition, we aimed to evaluate the correlation between regional amyloid deposition and local fALFF signals within cortical regions mainly affected by amyloid in the two high-risk groups.

## Materials and methods

### Participants

In this study, 18 T2DM participants were recruited from the university specialist clinic based on the American Diabetes Association (ADA) diagnostic criteria. All T2DM were cognitively normal with the local version of the Montreal Cognitive Assessment (HK-MoCA) score ≥ 26 (Wong et al., 2009). Eleven SCD participants were consecutively recruited from the local memory clinic of a university hospital during the period from June 2017 to June 2019. The final diagnosis of cognitively impaired subjects was made by a multi-disciplinary panel, consisting of a neuroradiologist (HKFM) and two geriatricians (YFS, PKCC). The panel made the clinical diagnosis of SCD according to Jessen et al. (2014a). A total of 18 healthy elderlies were recruited from community centers that met the inclusion criteria involving HK-MoCA score≥26 and normal pressure <140/90 mmHg. Healthy elderlies with prediabetes, diabetes, claustrophobia, and previous cerebrovascular diseases were not included. For all subjects, the exclusion criteria included a history of stroke, head injury, seizure, migraine, cancer within 5 years, active infection, renal or other organ failures, psychiatric illness, regular alcohol or drug abuse, deafness, or other physical barriers.

All participants underwent clinical evaluation, neuropsychological test, structural-MRI (sMRI), and rs-fMRI scanning. The SCD and T2DM groups were also assessed by 18F-Flutemetamol PET. The duration between MRI and amyloid PET scanning was within 1 week. The determination of amyloid positivity of 18F-Flutemetamol PET was dependent on visual rating by a neuroradiologist (HKFM) who had successfully trained through an electronic training program developed by GE Healthcare (Buckley et al., 2017). Informed consent was obtained from all non-demented participants. The study logistics complied with the Declaration of Helsinki and ethical approval of the research protocol had been obtained from the Institutional Review Board of the University of Hong Kong and the Hospital Authority Hong Kong West Cluster.

### Clinical and neurological assessment

All participants underwent HK-MoCA (Wong et al., 2009) as a neuropsychological test by a trained research assistant. For the history of vascular risk factors (such as hypertension, hyperlipidemia) and comorbidities, information of all participants was collected from their medical history recorded in a clinical management system database or interviews managed by a research nurse.

### Data acquisition

#### MRI acquisition

MR images were acquired by a 3T clinical scanner (Philips Healthcare, Achieva) using a 32-channel head coil at the university imaging center. MRI sequences with parameters as follows: Three-dimensional (3D) T1-weighted MPRAGE using repetition time (TR) = 6.8 ms, echo time (TE) = 3.2 ms, thickness = 1.2 mm, flip angle = 8°, field of view (FOV) = 256 × 240 × 204 (mm), matrix = 256 × 240; 3D FLAIR using TR = 6.8 ms, TE = 3.2 ms, thickness = 1.2 mm, field of view (FOV) = 250 × 250 × 184 (mm), matrix = 208 × 207. rs-fMRI with a gradient-echo echo-planar sequence with TR/TE = 2,000/30 ms, flip angle = 90, FOV = 230 × 230 × 144 (mm), image acquisition resolution = 3.28 × 3.28 mm^2^, slice thickness = 4 mm, number of volumes = 180. During rs-fMRI, participants were instructed to focus on a cross in the mirror and not to think of anything in particular.

#### ^18^F-flutemetamol PET-CT imaging acquisition

All participants were required to fast for at least 6 h and rest in a dimmed room waiting for a tracer injection. A bolus of 18F-flutametamol was administered intravenously (within 40 s) to the patients at a dosage of 185Mbq (~5mCi). The scanning started at 90 min after injection, using an integrated in-line PET/CT scanner with 3D list mode. Filtered back-projection reconstruction was used with a slice thickness of 2–4 mm, matrix size of 128*128 with a pixel size of 2 mm. A full width half-maximum (FWHM) post-smoothing filter was applied, of not more than 5 mm. The duration of the scan lasted 30 min (Nelissen et al., 2009; Vandenberghe et al., 2010).

### White matter lesions (WMLs) quantification

Periventricular and subcortical WMLs were quantified by Fazekas score (0–3; Fazekas et al., 1987) through axial FLAIR images. The scoring was performed and confirmed by trained personnel.

### Imaging processing and data extraction

#### 18F-flutemetamol PET processing in cortex ID ROIs

The post-processing of fused structural MRI and 18F-Flutemetamol images was performed by semi-automatic commercially available software (Cortex ID software, GE Healthcare Ltd., USA) for all SCD and T2DM participants. The procedures included realignment, co-registration, and normalization. In addition, a quantitative analysis of 1 global and 16 region-of-interests (ROIs) was made by Cortex ID software, including bilateral prefrontal, anterior cingulate, precuneus/posterior cingulate, parietal, lateral temporal, occipital, sensorimotor, and mesial temporal regions. Normalized for injected dose and body weight of each subject, standardized uptake values (SUVs) were calculated in all regions. The standardized uptake value ratio (SUVR) was the ratio between SUV data of the target region and reference region (pons in our study).

As Palmqvist et al. reported, the posterior cingulate cortex, precuneus, rostral anterior cingulate cortex, medial, and lateral orbitofrontal cortex are most prone to the earliest accumulation of Aβ (Palmqvist et al., 2017). These regions also correspond to those involved at stage 1 or 2 according to in vivo staging amyloid scheme (Grothe et al., 2017). Part of the regions (bilateral prefrontal, anterior cingulate, precuneus/posterior cingulate) used in this study is characteristic of being affected by Aβ at an early stage. Besides, the regions late affected by amyloid were also involved, such as the occipital lobe and mesial temporal lobe, which were closely associated with vision and memory respectively and frequently addressed in T2DM (Xia et al., 2013; Cui et al., 2014; Wang et al., 2014) and cognitive impairment diseases (Han et al., 2011; Sun et al., 2016; Zhang et al., 2021).

#### 18F-flutemetamol PET processing and SUVR extraction in survived clusters

Structural MRI images and 18F-Flutemetamol PET images underwent re-origin and realignment by Statistical Parametric Mapping (SPM) 12 implemented in MATLAB R2019a. The resultant PET images were co-registered with the corresponding sMRI images. The fused images were normalized and smoothed by SPM12. The masks of survived clusters with differences in HC vs. T2DM and HC vs. SCD were saved and applied to fused images for obtaining the SUV values within each cluster by ROI signal extractor function based on DPARSFA (Data Processing Assistant for Resting-State fMRI advanced version) toolbox (http://rfmri.org/DPARSF, version 5.3), implemented within MATLAB R2019a (Yan and Zang, 2010). All cluster-based SUV values were normalized to pons' SUV value to obtain SUVR values for every participant.

#### Rs-fMRI processing and fALFF extraction in cortex ID ROIs and survived clusters

Structural MRI images and rs-fMRI images were processed by DPARSFA. The sMRI image taken for an individual is the first realigned and followed by a reorientation, bet, co-registration, and segmentation. The rs-fMRI image underwent realignment and co-registered with sMRI images. The fused rs-fMRI underwent normalization and smoothing with a Gaussian kernel size of 4^*^4^*^4 mm^3^ full-width half-maximum (FWHM). In addition, a linear tread model was applied to avoid the systematic increased or decreased signals with time (Lowe and Russell, 1999), and a rigid-body 6 head motion model was selected in this study to reduce the non-neuronal blood-oxygen-level-dependent (BOLD) fluctuations and effects of head motion (Fox et al., 2005; Kelly et al., 2008). After that, fALFF maps were obtained from fused rs-fMRI images. The fALFF of each voxel was normalized by the global mean fALFF within a brain mask to obtain standardized fALFF (mfALFF). Therefore, the following fALFF data was obtained from individuals' mfALFF maps. The WFU_pickatlas toolbox (http://www.fmri.wfubmc.edu/cms/software, version 3.0.5; Maldjian et al., 2003) and ROI signal extractor function of DPARSFA contributed to creating the masks of ROIs and signal extraction, respectively.

The generated masks of ROIs were the same as those made by Cortex ID software (GE Healthcare Ltd., USA), including bilateral prefrontal, anterior cingulate, precuneus/posterior cingulate, parietal, lateral temporal, occipital, sensorimotor, and mesial temporal regions. Extraction of the same regional values in rs-fMRI and 18F-Flutemetamol images was for further correlation analysis. Besides, the masks of survived clusters with altered fALFF signals, when compared T2DM and SCD groups to the HC group, were also saved and applied to rs-fMRI images for obtaining the signal of fALFF of each cluster.

### Statistical analysis

All statistical tests were performed on SPSS software (SPSS version 23.0.0, Chicago, IL, USA). Shapiro-Wilk test was used to check the normality of data. Our data were not normally distributed. Among three groups, the comparison of continuous data (age, MoCA scores) and ordinal data (WMLs) was assessed by the non-parametric independent-samples Kruskal–Wallis test, and the categorical difference (sex and vascular risk factors) was assessed by the Chi-square test. The comparison of quantitative amyloid deposition was assessed by a non-parametric Kolmogorov–Smirnov test between T2DM and SCD groups. In T2DM and SCD groups, the correlations between regional standardized fALFF signals and amyloid burden in SUVR extracted from Cortex ID ROIs and survived clusters were evaluated by a non-parametric partial correlation test controlling for age and sex. All *p*-values were two-sided and set at 0.05.

### Voxel-wise analysis

All voxel-wise tests were implemented on the DPARSFA toolbox (statistical analysis section). The analyses of standardized fALFF maps in a voxel-wise manner were performed within a gray matter mask. In addition, the Gaussian random field (GRF) method was applied for multiple comparison corrections. The threshold was set at voxel *p* < 0.01, cluster *p* < 0.05 and a cluster size > 55 voxels. All *p-*values were two-tailed.

### Voxel-wise fALFF analysis within each group

To detect the standardized fALFF patterns in each group, a one-sample *t*-test was applied through a voxel-by-voxel analysis. Standardized fALFF maps of each group were imported and set the base as 1, which indicated that compared to the mean global fALFF maps of each group.

### Voxel-wise fALFF analysis between groups

For assessing the differences in standardized fALFF signals between two groups (SCD vs. HC and T2DM vs. HC), a two-sample *t*-test was performed in a voxel-wise manner. Age, sex, WMH, and vascular risk factors (hyperlipidemia and hypertension) were input as covariates. Modulated gray matter (GM) maps obtained from DPARSF (segmentation section) were also used as a covariate to avoid the possible effects of GM volume differences.

## Results

### Demographic characteristics, neuropsychological characteristics, and regional amyloid deposition of the cohort

Table 1 showed the demographic characteristics and neuropsychological characteristics of our cohort including WMLs, vascular risk factors, and comorbidities of our cohort. The mean age in the T2DM group was younger (age: 62.50 ± 3.65), than SCD (70.91 ± 7.40) and HC groups (age: 71.33 ± 5.64) with *p* < 0.01 and *p* < 0.001 respectively. In addition, the distribution of sex among the three groups was different with *p* < 0.0001. The comparable mean MoCA score in HC (27.56 ± 1.04), T2DM (26.50 ± 2.07), and SCD (25.89 ± 6.99) indicated intact cognition. Our T2DM subjects had ~20.06-year with standard deviation (SD) = 8.50 of DM duration.

**Table 1:** Demographic and neuropsychological characteristics of the cohort.

**Variable**		**Group**		
		**T2DM**	**SCD**	**HC**
		***n*** **=** **18**	***n*** **=** **11**	***n*** **=** **18**
Age^a^, mean (SD)		62.50 (3.65)	70.91 (7.40)	71.33 (5.64)
Sex^b^, No.	F	2	8	16
	M	16	3	2
Disease duration, mean (SD)		20.06 (8.50)	/	/
MoCA score, mean (SD)		27.56 (1.04)	25.89 (6.99)	26.50 (2.07)
Amyloid positivity, No. (%)		1 (6%)	2 (18%)	/
Mean global amyloid deposition in SUVR (SD)		0.46 (0.07)	0.49 (0.07)	/
WMLs (Fazekas score), Median	Periventricular	0	1	0
	Subcortical	1	1	1
Vascular risk factors, No. (%)	Hypertension	9 (50%)	3 (27%)	8 (44%)
	Hyperlipidemia^c^	16 (89%)	3 (27%)	4 (22%)
	Hypertension + hyperlipidemia	9 (50%)	1 (9%)	4 (22%)
Comorbidities, No. (%)	Retinopathy	5 (28%)	/	/
	Retinopathy + maculopathy	2 (11%)	/	/
	Retinopathy + nephropathy	1 (6%)	/	/

^a^Independent-samples Kruskal-Wallis Test in age, p < 0.01 in SCD vs. T2DM; p < 0.001 in HC vs. T2DM.

^b^Chi-square test with p < 0.0001 in sex among three groups.

^c^Chi-square test with p < 0.0001 in hyperlipidemia among three groups.

The 18F-Flutemetamol-PET results revealed 1 T2DM (positive prevalence: 6%) and 2 SCD participants (positive prevalence: 22%) had positive scanning. The mean global SUVR value was 0.46 (SD = 0.07) and 0.49 (SD = 0.07) in T2DM and SCD groups, respectively, that lower than the threshold (SUVR of 0.62) used for defining positive amyloid binding at the global level (Thurfjell et al., 2014). Three groups presented the same median of 1 in subcortical white matter hyperintensity. The median in periventricular was the same in T2DM and HC groups (median = 0), and higher in the SCD group (median = 1). The distribution of WMLs had no significant difference in ternary comparison.

Referring to vascular risk factors, 50% of T2DM, 27% of SCD, and 44% of HC participants had hypertension. The proportion of subjects with hyperlipidemia was up to 89% in the T2DM group which was higher than that in the HC group (22%) and SCD group (27%) with a *p* < 0.0001. There were 50% of T2DM participants, 9% of SCD participants, and 44% of HC had two vascular risk factors. Additionally, five participants with retinopathy were found to have T2DM (28%). Three of them had two comorbidities recorded as retinopathy with maculopathy (2, 11%) or retinopathy with nephropathy (1, 6%).

Quantitative regional amyloid deposition in SCD and T2DM groups and comparisons between groups are shown in Table 2. There was no significant difference in amyloid deposition shown between groups as *p* > 0.05 were presented in all regions.

**Table 2:** The mean values of regional amyloid deposition in SCD and T2DM.

**ROIs**	**SCD**		**T2DM**		**SCD vs. T2DM**
	**Mean**	**SD**	**Mean**	**SD**	***P*** **-value**
Prefrontal R	0.45	0.07	0.42	0.06	>0.05
Prefrontal L	0.46	0.09	0.42	0.08	>0.05
Anterior Cingulate R	0.49	0.07	0.46	0.07	>0.05
Anterior Cingulate L	0.53	0.08	0.50	0.08	>0.05
Precuneus/Posterior Cingulate R	0.49	0.07	0.47	0.08	>0.05
Precuneus/Posterior Cingulate L	0.52	0.09	0.49	0.10	>0.05
Parietal R	0.50	0.07	0.47	0.08	>0.05
Parietal L	0.48	0.08	0.45	0.09	>0.05
Temporal Lateral R	0.54	0.05	0.51	0.05	>0.05
Temporal Lateral L	0.53	0.07	0.50	0.07	>0.05
Occipital R	0.55	0.07	0.51	0.04	>0.05
Occipital L	0.56	0.08	0.52	0.05	>0.05
Sensorimotor R	0.46	0.05	0.44	0.06	>0.05
Sensorimotor L	0.47	0.04	0.44	0.07	>0.05
Temporal Mesial R	0.50	0.02	0.52	0.04	>0.05
Temporal Mesial L	0.49	0.02	0.51	0.04	>0.05

L, Left; R, Right.

### Rs-fMRI fALFF

#### fALFF pattern in each group

**Figure 1:**
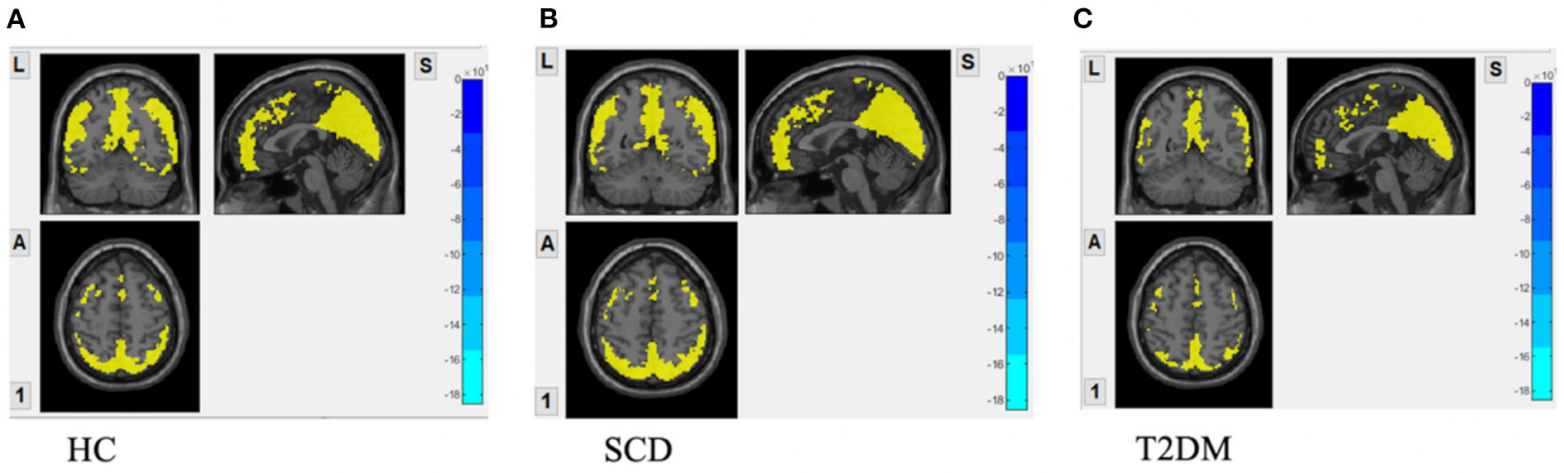
fALFF maps with activation pattern compared to global fALFF maps assessed by one sample *t*-test. **(A)** Increased fALFF pattern in HC group; **(B)** increased fALFF pattern in SCD group; **(C)** increased fALFF pattern in DM group GRF correction was applied with voxel *p*-value < 0.01, cluster *p*-value < 0.05 and a cluster size > 55 voxels.

In Figure 1, one-sample t-test results of fALFF maps of the three groups are shown. The significantly increased fALFF signals compared to global fALFF signals were mainly concentrated within DMN including bilateral precuneus cortex and middle occipital lobe for all groups. The elevated signals in the calcarine, cuneus cortex, and inferior frontal lobe were also observed. In HC and SCD groups (Figures 1A,B), the activated fALFF signals were exhibited additionally in the bilateral parietal lobe, inferior occipital lobe, middle temporal lobe, angular lobe, and posterior part of the cerebellum.

#### fALFF comparison between HC and T2DM groups

**Figure 2:**
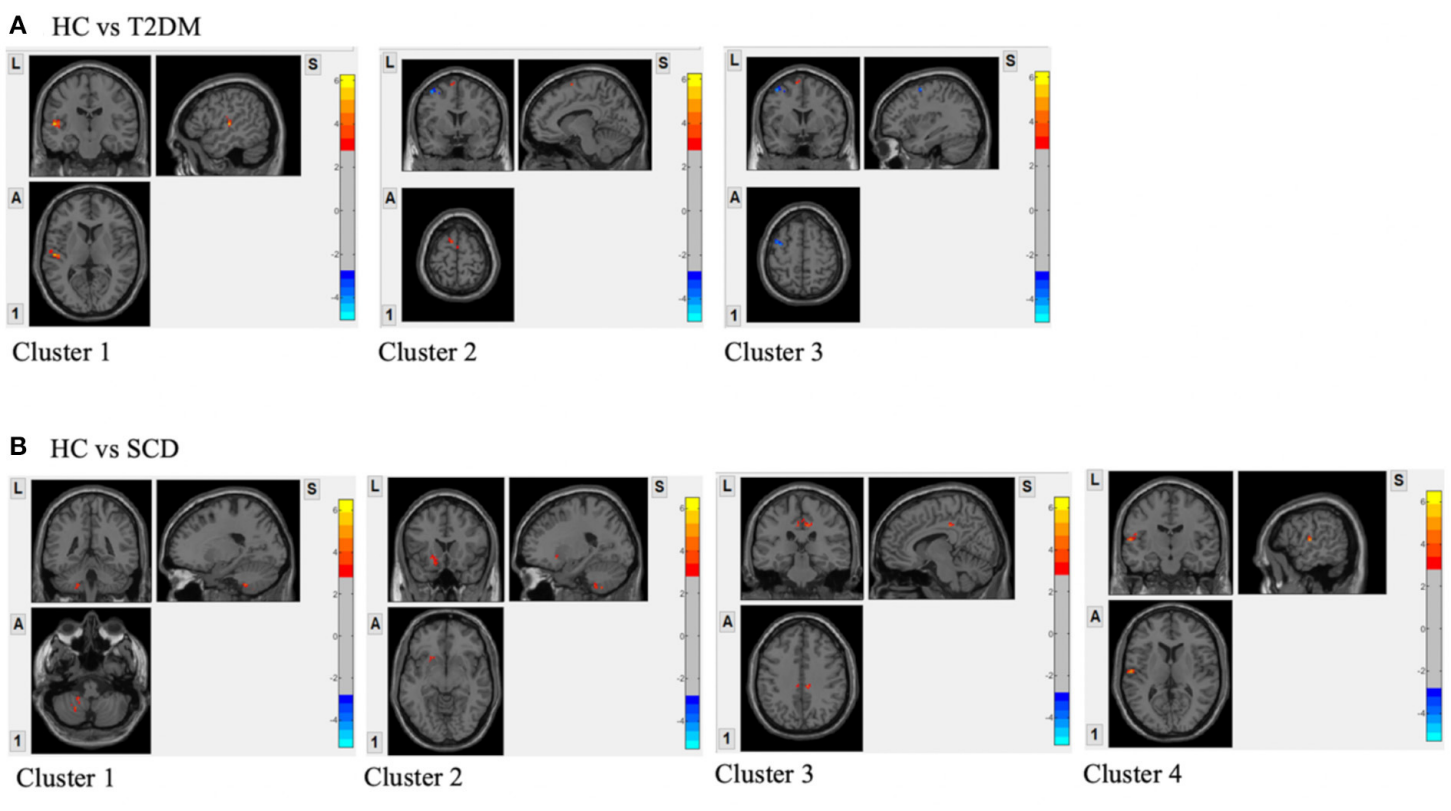
**(A)** Regions with the significant difference in activation between HC and T2DM assessed by two-sample *t*-test; **(B)** Regions with the significant difference in activation between HC and SCD assessed by two-sample *t*-test. GRF correction was applied with voxel *p*-value < 0.01, cluster *p*-value < 0.05 and a cluster size > 55 voxels; Covariates included sex, age, WMLs and vascular risk factors.

Compared to the HC group, T2DM participants had increased fALFF signals in two clusters (Figure 2A). Cluster 1 included 102 voxels and 79% of the voxels within this cluster belong to the left superior temporal gyrus (Temporal_Sup_L). Cluster 2 with 56 voxels consisted of the left supplementary motor area (Supp_Motor_Area_L) only. The decreased fALFF signal was shown in cluster 3 including the left precentral gyrus (Precentral_L) and left middle frontal gyrus (Frontal_Mid_L) occupying 59% and 41%, respectively (Table 3).

**Table 3:** Clusters showing fALFF difference in HC vs. T2DM and HC vs. SCD with cluster size and MNI coordinates.

**fALFF signals**	**Cluster position** **(decided by predominant voxels)**	**Cluster size** **(No. of voxels, volume in mm3)**	**Peak coordinate** **(in MNI space)**	**Peak intensity** **(*****T*****-value)**	**Cluster breakdown** **(No. of voxels, % in cluster)**
T2DM>HC	Cluster 1: L Temporal lobe	102, 816	X = −52, Y = −18, Z = 8	5.8813	**Temporal_Sup_L (81, 79%)**
					Temporal_Mid_L (9, 9%)
					Heschl_L (6, 6%)
					Rolandic_Oper_L (4, 4%)
					Postcentral_L (2, 2%)
	Cluster 2: L Frontal lobe	56, 448	X = −10, Y = 4, Z = 68	3.6728	**Supp_Motor_Area_L (56, 100%)**
T2DM < HC	Cluster 3: L Frontal lobe	63, 504	X = −36, Y = 4, Z = 58	−2.761	**Precentral_L (37, 59%)**
					Frontal_Mid_L (26, 41%)
SCD>HC	Cluster 1: L Cerebellum	80, 640	X = −20, Y = −42, Z = −46	4.6196	**Cerebellum_8_L (57, 71%)**
					Cerebellum_9_L (20, 25%)
					Cerebelum_1_L (2, 3%)
					Cerebellum_7b_L (1, 1%)
	Cluster 2: L Central structures	73, 584	X = −18, Y = 14, Z = −2	4.1051	**Putamen_L (33, 45%)**
					Rectus_L (18, 25%)
					Frontal_Sup_Orb_L (7, 10%)
					Olfactory_L (4, 5%)
					Pallidum_L (3, 4%)
					Caudate_L (2, 3%)
					Insula_L (1, 1%)
	Cluster 3: R Cingulate gyrus	64, 512	X = 8, Y = −28, Z = 38	4.2046	**Cingulum_Mid_R (43, 67%)**
					Cingulum_Mid_L (20, 31%)
					None (1, 2%)
	Cluster 4: L Temporal lobe	63, 504	X = −58, Y = −14, Z = 10	4.9823	**Temporal_Sup_L (27, 43%)**
					Rolandic_Oper_L (13, 21%)
					Postcentral_L (12, 19%)
					Heschl_L (11, 17%)

Temporal_Sup_L, left superior temporal gyrus; Temporal_Mid_L, left middle temporal gyrus; Heschl_L, left heschl gyrus; Rolandic_Oper_L, left rolandic operculum; Postcentral_L, left postcentral gyrus; Supp_Motor_Area_L, left supplementary motor area; Precentral_L, left precentral gyrus; Frontal_Mid_L, left middle frontal gyrus; Cerebellum_8_L, left cerebellum crus 8; Cerebellum_9_L, Cerebelum_1_L, left cerebellum crus 1; Cerebellum_7b_L, left cerebellum crus 7; Putamen_L, left putamen; Rectus_L, left rectus gyrus; Frontal_Sup_Orb_L, left superior frontal gyrus, orbital; Olfactory_L, left olfactory cortex; Pallidum_L, left pallidum; Caudate_L, left caudate nucleus; Insula_L, left insula; Cingulum_Mid_R, right cingulate gyrus, mid part; Cingulum_Mid_L, left cingulate gyrus, mid part. The bold values indicate the cluster position with maximum voxel.

#### fALFF comparison between HC and SCD groups

In Figure 2B, four clusters were shown to have increased fALFF signals in the SCD group compared to the HC group. Cluster 1 with 80 voxels was in the left cerebellum and 71% of the cluster was within left cerebellum crus 8 (Cerebellum_8_L). The rest three clusters had 73, 64, and 63 voxels. The voxels in the left putamen (Putamen_L; 33, 45%), right cingulate gyrus, mid part (Cingulum_Mid_R; 43, 67%), and left superior temporal gyrus (Temporal_Sup_L; 27, 43%) occupied the highest proportion in each cluster (Table 3).

### Correlation analysis

#### Partial correlation in T2DM and SCD groups: Survived clusters-based

Table 4 showed no significant correlation shown between regional fALFF values and local amyloid deposition in the survived clusters for each group.

**Table 4:** Non-parametrical partial correlation (control for age and sex) between regional fALFF values and local amyloid deposition in T2DM and SCD groups, survived clusters-based.

**Group**	**Clusters**	***R*** **-value**	***P*** **-value**
T2DM	Cluster1	0.108	0.691
	Cluster2	0.161	0.552
	Cluster3	0.3	0.26
SCD	Cluster1	−0.626	0.071
	Cluster2	0.066	0.867
	Cluster3	0.041	0.916
	Cluster4	−0.02	0.959

#### Partial correlation in T2DM and SCD groups: Cortex ID ROI-based

A non-parametric partial correlation controlled for age and sex between 16 regional fALFF values and local amyloid deposition was performed for the T2DM and SCD groups. In Table 5, fALFF values of the left anterior cingulate cortex (ACC) were negatively correlated with local amyloid burden at a significant level (*r* = −0.778, *p* < 0.001). In contrast, fALFF values were positively correlated with amyloid deposition in the right occipital lobe (*r* = 0.804, *p* < 0.001) and left mesial temporal lobe (*r* = 0.604, *p* < 0.05). In the SCD group (Table 6) significant negative correlation was shown in the left ACC (*r* = −0.755, *p* < 0.05).

**Table 5:** Non-parametrical partial correlation (control for age and sex) between regional fALFF values and local amyloid deposition in T2DM group, cortex ID ROI-based.

**T2DM**	***R*** **-value**	***P*** **-value**
Prefrontal R	−0.365	0.164
Prefrontal L	−0.361	0.17
Anterior Cingulate R	−0.362	0.168
Anterior Cingulate L	**−0.778**	**<0.001**
Precuneus/Posterior Cingulate R	0.206	0.444
Precuneus/Posterior Cingulate L	0.19	0.482
Parietal R	0.137	0.612
Parietal L	0.289	0.277
Temporal Lateral R	0.249	0.353
Temporal Lateral L	0.056	0.836
Occipital R	**0.804**	**<0.001**
Occipital L	0.149	0.106
Sensorimotor R	0.345	0.191
Sensorimotor L	0.295	0.267
Temporal Mesial R	0.369	0.159
Temporal Mesial L	**0.604**	**<0.05**

L, Left; R, Right. The bold values indicate R-value with P-value < 0.05.

**Table 6:** Non-parametrical partial correlation (control for age and sex) between regional fALFF values and local amyloid deposition in SCD group, cortex ID ROI-based.

**SCD**	***R*** **-value**	***P*** **-value**
Prefrontal R	0.409	0.274
Prefrontal L	0.189	0.626
Anterior Cingulate R	−0.605	0.084
Anterior Cingulate L	**−0.755**	**<0.05**
Precuneus/Posterior Cingulate R	−0.343	0.367
Precuneus/Posterior Cingulate L	−0.11	0.778
Parietal R	−0.145	0.71
Parietal L	0.003	0.994
Temporal Lateral R	0.266	0.49
Temporal Lateral L	−0.143	0.714
Occipital R	−0.178	0.646
Occipital L	−0.123	0.752
Sensorimotor R	−0.514	0.157
Sensorimotor L	−0.413	0.269
Temporal Mesial R	−0.641	0.063
Temporal Mesial L	−0.503	0.168

L, Left; R, Right. The bold values indicate R-value with P-value < 0.05.

## Discussion

### Altered fALFF signals in T2DM

The increased fALFF signals in the T2DM group displayed in the left superior temporal gyrus and left supplementary motor areas. The superior temporal gyrus within the auditory network (Beckmann et al., 2005) plays an essential role in language, speech, and auditory processing (Bueti et al., 2008; Patel et al., 2021). A previous study stated that impaired language processing was associated with the superior temporal lobe during dysglycemia (Allen et al., 2015). Consistent with the Zhou et al. study, hyperactivity in the left superior temporal gyrus was observed when comparing T2DM with MCI and HC (Zhou et al., 2014). The increased neural activity was also shown in the left supplementary motor area, a part of the sensorimotor network (Beckmann et al., 2005). The supplementary motor area is responsible for controlling language and speech processing (Hertrich et al., 2016), as well as complex movement planning and coordination (Hiroshima et al., 2014). However, study by Wang et al. illustrated that there were decreased ALFF signals in the left supplementary motor area, as shown in the T2DM group compared to HC (Wang et al., 2014). The discrepancy may depend on the different cognitive levels in the T2DM participants between that study and the current one since cognitive decline was associated with abnormal ALFF signals in particular regions (Xia et al., 2013; Cui et al., 2014; Wang et al., 2014). The increased fALFF signals in the superior temporal gyrus may be associated with compensation counterbalancing the possible decline in language, speech, or auditory processing. Similarly, hyperactivity in the supplementary motor area may make up for motor deficits.

In the meantime, the cluster with decreased fALFF signals was shown in the left precentral gyrus. The region contains the primary motor cortex (Lemon, 2008) which contributes to controlling voluntary motor movement on the contralateral side of the body (Cross et al., 2020). Although motor dysfunction is usually linked to T2DM *via* diabetic peripheral neuropathy (DNP; Fulk et al., 2010; Gorniak et al., 2014, 2019; Ochoa et al., 2016), DNP is not the sole contributor to motor dysfunction (Fulk et al., 2010; Hewston and Deshpande, 2016). The subtle diabetic-related decline in sensory, metabolic muscle, and executive functions could also contribute to impaired balance in T2DM (Hewston and Deshpande, 2016). Besides, in previous studies, the decreased fALFF in the frontal lobe was significantly correlated with severe microvascular disease (Wang et al., 2014), and the decreased CBF in this region was also found in the T2DM group with a higher incidence of vascular risk factors (Last et al., 2007; Chau et al., 2020). Consistently, our T2DM group had a much higher prevalence of hyperlipidemia (89%) compared to the HC group (22%). Although both the supplementary motor area and precentral gyrus are parts of the frontal lobe and within the sensorimotor network, the supplementary motor area plays an important role in internal-guided movement whilst the precentral gyrus contributes to both internal and external-guided movements (Ninomiya et al., 2019). This could partly explain the opposite neural activity change in the two sites.

### Altered fALFF signals in SCD

Compared to HC, the areas showed increased fALFF signals in the SCD group included the left posterior cerebellum, left superior temporal gyrus, left putamen, and right middle cingulate gyrus extending to the paracingulate gyrus. The cerebellum has an important role in motor coordination and modulation of cognition and emotion (Jacobs et al., 2017). As mentioned before, the superior temporal lobe contributes to language processing (Bueti et al., 2008; Patel et al., 2021). Language decline is a common feature (Forbes-McKay et al., 2013) in AD that is linked to the severity of the disease (Kav and Dassa, 2018). The increased ALFF/fALFF signals in the superior temporal gyri and cerebellum were also reported by previous studies when comparing SCD with HC (Sun et al., 2016) and comparing amnestic MCI with HC (Han et al., 2011; Yin et al., 2014). The putamen is a part of the basal ganglia that regulates a wide range of motor and cognition (Nagano-Saito et al., 2014). One previous work proved the decreased volume of putamen (De Jong and van der Grond, 2008), which may be associated with cognitive impairment. The middle cingulate and paracingulate gyri house Brodmann's area 23 and are located in the ventral part of the posterior cingulate cortex (PCC; Cera et al., 2019). PCC is a core component of the DMN that contributes to visuospatial orientation, body navigation (Vogt, 2005), self-reflection, and autobiographical memories (Spreng et al., 2009). There is an abundance of converging evidence on altered DMN connectivity in SCD (Dillen et al., 2017; Xue et al., 2019), MCI (Jacobs et al., 2013; Wang et al., 2013a; Xue et al., 2019), and AD (Toussaint et al., 2014; Dai et al., 2015; Dillen et al., 2017; Xue et al., 2019). Furthermore, the increased neural activity in the part of temporal, occipital, parietal, and subcortical regions was negatively correlated with various neuropsychological tests' performance in the SCD group (Zhang et al., 2021). With this finding, we may speculate that the increased local neural activity in SCD may represent a compensatory mechanism that counterbalances neural damage and functional decline.

The results reported by the previous meta-analytic studies may illustrate the altered spontaneous activity in patients with high-risk factors more fairly. Xia et al. observed reduced brain activity in the bilateral lingual gyrus, left postcentral gyrus, right inferior temporal gyrus, right cerebellar culmen, right insula, and right posterior cingulate cortex but hyperactivity in the left superior frontal gyrus and right precuneus, when comparing T2DM group with HC group (Xia et al., 2017). In another meta-analytic study reported by Liu et al. (2021), regional hypoactivity in patients with T2DM was presented in the right medial superior frontal gyrus, right superior temporal gyrus, and left lingual gyrus, and hyperactivity was shown in the right cerebellum. Several key regions were consistently reported in previous meta-analytic studies and the current study, for instance, the superior frontal gyrus and superior temporal gyrus, although the specific alterations (increased or decreased brain activity) may not be accordant. The heterogeneity of included studies and different tools for data analysis may result in the discrepancy. Similarly, in a previous meta-analytic study investigating spontaneous brain activity in MCI, decreased ALFF values were found in the bilateral PCC (Pan et al., 2016). Our SCD group showed increased fALFF values in the ventral part of PCC. Moreover, the elevated Aβ deposition and decreased glucose metabolism of this region were also observed in MCI and AD (He et al., 2014). PCC is a major component within the DMN and is prone to amyloid deposition at the early stage of AD, which could be a key region for early diagnosis of AD. In addition, our study demonstrated more regions showing increased brain activity compared to previous studies. The hyperactivity in these regions could be closely associated with the intact cognition of SCD and T2DM groups (showing comparable regional amyloid load in Table 2), such as making up for possible deficits to compensate for the loss of cognitive function by recruiting additional neural activities.

### Partial correlation in T2DM and SCD groups: Survived cluster-based

The fALFF signals in the survived clusters were not correlated with the local amyloid load when comparing each high-risk group with HC. However, the correlation was established between two variates in certain pre-set regions in high-risk groups (Tables 4, 5) discussed below. The results are not against each other since they are two different comparisons. We hypothesized that altered fALFF in those survived clusters may not be solely dependent on local amyloid deposition. Cognitive impairment could be another factor affecting the correlation. Altered fALFF values from particular survived clusters were significantly correlated to MoCA scores for cognitively impaired patients with T2DM reported by one previous study. Nevertheless, our T2DM participants were cognitively normal. Besides, Chen et al. reported that enhanced topographical pro perties in the SCD group compared to the HC group were negatively correlated with cerebrospinal fluid (CSF) Aβ levels in those DMN-related regions (Chen et al., 2020). Notably, abnormal Aβ level detected by CSF maker was prior to amyloid plaques detected by amyloid-PET (Mattsson et al., 2015; Palmqvist et al., 2017) which may result in this difference.

### Partial correlation in T2DM and SCD groups: Cortex ID ROI-based

In both high-risk groups, fALFF signals were negatively correlated with amyloid deposition in the left ACC which is a part of the DMN and responsible for memory processing (Buckner et al., 2005; van den Heuvel and Hulshoff Pol, 2010). Previous research had proved that DMN connectivity dysfunction was associated with amyloid burden in the cognitively normal elderly (Sheline et al., 2010; Mormino et al., 2011; Wang et al., 2013b; Elman et al., 2016; Palmqvist et al., 2017). Moreover, in the Scheel et al. study, a robust negative correlation between ALFF signals and the amyloid load was predominantly in the DMN and visual network, in the amnesic MCI group (Scheel et al., 2021). The negative correlation shown in the ACC for both SCD and T2DM groups may illustrate the vulnerability of the ACC to amyloid pathology.

In T2DM, a positive correlation was also shown in the mesial temporal within the DMN and occipital lobes within the visual network. Compared to the ACC, the mesial temporal and occipital lobes affected by Aβ are commonly seen in the late stage of AD (Thal et al., 2002; Grothe et al., 2017). One mice model study showed that increased physiological neural activity was closely associated with early and soluble Aβ oligomers, before the formation of fibrillar Aβ plaques (Bero et al., 2011). Palmqvist et al. found that the connectivity within the DMN shifted from hyperconnection to hypoconnection when CSF Aβ_42_ levels dropped from near abnormal range to abnormal level (Palmqvist et al., 2017). Hence, we speculate that the specific correlation may be associated with the temporal difference between the regions affected by Aβ deposition and the local magnitude of Aβ abnormality. The occipital lobe has an important role in visual processing (van den Heuvel and Hulshoff Pol, 2010). Although amyloid accumulation in the visual network was observed to be less as compared to the DMN in the early stages of AD (Palmqvist et al., 2017), 28 and 11% of our T2DM participants with retinopathy and with both retinopathy and maculopathy, respectively, may contribute to the correlation. Atrophy of the mesial temporal regions was initiated at a very early stage (Cho et al., 2016), whereas the Aβ load in these regions was very low or even non-existent (Braak and Braak, 1991; Arriagada et al., 1992; Price and Morris, 1999). Consequently, local amyloid uptake may be difficult to detect or quantify due to atrophy. Therefore, the positive correlation in the mesial temporal regions requires further validation. A positive correlation was also seen between amyloid uptake and dynamic functional connectivity in cognitively unimpaired elderlies with normal amyloid-PET results (Hahn et al., 2019). The enhanced dynamic connectivity may reflect a compensation to maintain the normal cognitive performance in the presence of increasing Aβ accumulation during the early phase of AD (Hahn et al., 2019). Previous studies incorporating different modalities also illustrated this phenomenon, including enhanced neuronal activation (Elman et al., 2014) and gray matter volume (van Loenhoud et al., 2017) observed in the early stages of dementia. It is theorized to make up for Aβ-induced neuronal damage and structural change. Correspondingly, a significant positive correlation in our study may also reflect a compensatory mechanism, in the form of cognitive reserve, to counterbalance the increased Aβ burden.

From another aspect, previous work recruiting non-demented elderlies with normal amyloid-PET results found that the earliest Aβ accumulation started in the core regions of DMN (precuneus, posterior cingulate cortex, and orbitofrontal cortex) and was associated with the altered functional connectivity (Palmqvist et al., 2017; Hahn et al., 2019). Similarly, the current study illustrated significant correlations between Aβ and local neural activity in certain ROIs within the DMN and visual network in non-demented SCD and T2DM groups with mean negative amyloid retention as well. All the findings suggest that the very early Aβ deposition can be assessed in non-demented elderlies when global amyloid retention is still normal and has been associated with brain functional connectivity or local neural activity.

There are several limitations to this study. Our sample size was small. Nevertheless, it is a pilot study investigating the possible association between early Aβ accumulation and local neural activity in non-demented high-risk populations. Other clinical data could not be assessed, such as blood pressure, body mass index, and education. Specific neuropsychological tests were not performed, which limited further analysis between different domains of cognition and neural activity.

## Conclusion

Taken all together, compared to the HC group, the altered neural activity in the T2DM group was predominately presented in the superior temporal and motor cortex. In the meantime, the altered neural activity in the SCD group was in the cerebellum, subcortical central structure, cingulate gyrus, and temporal lobe. However, in both groups, altered fALFF signals of these regions were found to be independent of local Aβ deposition. Moreover, the very early amyloid uptake in the SCD and T2DM groups was observed to affect the local neural activity shown in several ROIs mainly involved in the DMN. The specific correlation (positive or negative) may be dependent on the temporal difference of the regions affected by Aβ deposition and the magnitude of local Aβ abnormality.

## Data availability statement

The raw data supporting the conclusions of this article will be made available by the authors, without undue reservation.

## Ethics statement

The study logistics complied with the Declaration of Helsinki and ethical approval of the research protocol had been obtained from the Institutional Review Board of the University of Hong Kong and the Hospital Authority Hong Kong West Cluster. The patients/participants provided their written informed consent to participate in this study.

## Author contributions

Y-WB contributed to conceptualization, data collection, data analysis, validation, visualization, and writing. Y-FS, PC, JK, FC, W-SC, and K-HC contributed to data investigation. HM contributed to conceptualization, data investigation, supervision, project administration, and funding acquisition. All authors contributed to the article and approved the submitted version.

## Funding

This work was supported by State Key Laboratory of Brain and Cognitive Sciences, The University of Hong Kong.

## Conflict of interest

The authors declare that the research was conducted in the absence of any commercial or financial relationships that could be construed as a potential conflict of interest.

## Publisher's note

All claims expressed in this article are solely those of the authors and do not necessarily represent those of their affiliated organizations, or those of the publisher, the editors and the reviewers. Any product that may be evaluated in this article, or claim that may be made by its manufacturer, is not guaranteed or endorsed by the publisher.
